# Learning symmetry-aware atom mapping in chemical reactions through deep graph matching

**DOI:** 10.1186/s13321-024-00841-0

**Published:** 2024-04-22

**Authors:** Maryam Astero, Juho Rousu

**Affiliations:** Computer Science, Aalto University, Konemiehentie 2, 02150 Espoo, Finland

**Keywords:** Atom mapping, Graph matching, Deep learning, Graph representation learning

## Abstract

Accurate atom mapping, which establishes correspondences between atoms in reactants and products, is a crucial step in analyzing chemical reactions. In this paper, we present a novel end-to-end approach that formulates the atom mapping problem as a deep graph matching task. Our proposed model, AMNet (Atom Matching Network), utilizes molecular graph representations and employs various atom and bond features using graph neural networks to capture the intricate structural characteristics of molecules, ensuring precise atom correspondence predictions. Notably, AMNet incorporates the consideration of molecule symmetry, enhancing accuracy while simultaneously reducing computational complexity. The integration of the Weisfeiler-Lehman isomorphism test for symmetry identification refines the model’s predictions. Furthermore, our model maps the entire atom set in a chemical reaction, offering a comprehensive approach beyond focusing solely on the main molecules in reactions. We evaluated AMNet’s performance on a subset of USPTO reaction datasets, addressing various tasks, including assessing the impact of molecular symmetry identification, understanding the influence of feature selection on AMNet performance, and comparing its performance with the state-of-the-art method. The result reveals an average accuracy of 97.3% on mapped atoms, with 99.7% of reactions correctly mapped when the correct mapped atom is within the top 10 predicted atoms.

**Scientific contribution**

The paper introduces a novel end-to-end deep graph matching model for atom mapping, utilizing molecular graph representations to capture structural characteristics effectively. It enhances accuracy by integrating symmetry detection through the Weisfeiler-Lehman test, reducing the number of possible mappings and improving efficiency. Unlike previous methods, it maps the entire reaction, not just main components, providing a comprehensive view. Additionally, by integrating efficient graph matching techniques, it reduces computational complexity, making atom mapping more feasible.

## Introduction

During a chemical reaction, reactant molecules are transformed into products. During this process, the bonds between atoms within the molecules are rearranged while the composition of the atoms remains unchanged. As a result, a precise and direct correspondence known as atom mapping, exists between the atoms in the reactants and those in the products. Atom mapping makes it possible to identify the reaction center [1], determine bond changes [2], assign reaction operators [3], extract reaction templates [4], identify optimal metabolic routes [5], and analyze scaffold transformations [6].

Traditional atom mapping methods can be categorized into two main categories: common substructure-based methods and optimization-based methods. Common substructure-based methods utilize algorithms to identify the maximum common substructure (MCS) and then employ post-processing steps to correct the remaining atoms that are not part of the MCS [7–10]. However, extracting the MCS is recognized as an NP-hard problem. On the other hand, optimization-based approaches focus on minimizing the number of bonds formed and broken during a reaction [11–15]. Some recent studies have emerged that combine both methods [16–18]. These methods have limitations when it comes to the efficiency and accuracy of handling complex reactions, which have driven researchers to explore deep learning based approaches for atom mapping.

In recent years, with increased data availability and computational power, deep learning approaches have shown promising results in addressing the atom mapping problem. A recent benchmarking study [19] has compared the performance of several existing atom mapping methods. This study has shown that RXNMapper [20], a data-driven method that was built over a transformer neural network architecture [21], outperforms other methods. RXNMapper utilizes the simplified molecular-input line-entry system (SMILES) representation for molecules. Utilizing an attention-guided approach, it maps the primary component of product atoms to reactant atoms, learning atom correspondence through attention weights derived from BERT (Bidirectional Encoder Representations from Transformers) [22], eliminating the need for labeled data during training. Subsequently, another noteworthy study introduced GraphormerMapper [23], a method that integrates a graph-based transformer with transformers to achieve atom mapping. The process of atom mapping begins by incorporating SMILES embeddings, degree of centrality, and pairwise atom distance to generate molecule embeddings. These embedded molecules are then inputted into a BERT model to learn atom relations within reactions. The identification of atom correspondences is achieved by averaging attention weights.

RXNMapper and GraphormerMapper, while showcasing strengths in addressing atom mapping challenges, exhibit certain limitations. Firstly, both methods do not consider molecule symmetry. Due to molecule symmetry, it is possible that a single chemical reaction has multiple valid atom mappings. Understanding and accounting for atoms with the same chemical environment and identical properties, known as topologically equivalent atoms [24], are essential steps in ensuring accurate and meaningful comparisons of atom mappings. Furthermore, RXNMapper’s unsupervised nature demands a vast dataset of unlabeled chemical reactions to capture intricate relationships in complex reactions. Additionally, mapping the main component of the product atoms to reactant atoms and reordering atoms makes it difficult to compare the predicted atom mapping with ground truth and use it on downstream tasks. On the other hand, GraphormerMapper’s efficacy depends on the quality of SMILES embeddings, introducing a potential limitation if these embeddings fail to accurately capture molecular nuances. Moreover, the combined complexity of graph-based and standard transformers in GraphormerMapper poses computational challenges.

To mitigate these issues, we take a different direction in this work to tackle the atom mapping problem by casting it as a graph matching problem. Graph matching is the process of identifying an optimal mapping between the nodes of two graphs. The goal of graph matching is to establish a mapping between nodes in the source graph and nodes in the target graph that maximizes the similarity between the corresponding nodes in the two graphs. Node similarity in graph matching can be computed using various similarity measures, including dot product and cosine similarity. These measures assess the similarity between nodes based on attributes or features associated with them [25].

Our proposed method utilizes deep learning models for graph matching to identify similarities between atoms based on their features [26–28]. Learning graph matching is the process of finding a model that can predict a match between two pairs of graphs from data [26, 29–31]. A fundamental tool for extracting meaningful affinities from graphs is the application of graph neural networks (GNNs), which are well-suited for handling graph-structured data and capturing complex relationships between nodes [32]. GNNs enable us to efficiently find the mapping between reactant and product atoms, thereby facilitating accurate atom mapping in chemical reactions.

The contributions of this paper can be summarized as follows:

- Proposing an end-to-end deep graph matching model for atom mapping: Our proposed model processes molecular graphs directly. This graph-based representation harnesses the structural characteristics of molecules, including atom and bond properties, making it well-suited for the analysis of chemical reactions.
- Enhancement of atom mapping accuracy through symmetry detection: We adapt the Weisfeiler-Lehman test to improve the accuracy of predicted atom mapping by incorporating molecular symmetry detection. This approach reduces the number of possible mappings, leading to enhanced accuracy and efficiency in atom mapping.
- Fully mapped atom mapping model by considering the whole atoms in reactions: Our proposed method maps the entire reaction, not just the main components in the reactant or product.
- Reduced computational complexity: Through the integration of efficient graph matching techniques and symmetry consideration strategies, our model mitigates the computational complexities typically associated with atom mapping.

## Atom mapping through deep graph matching

### Atom mapping problem

Atom mapping of chemical reactions refers to the process of tracking and assigning direct connections between atoms in the reactant molecules and their corresponding atoms in the product molecules. This one-to-one correspondence provided by atom mapping enables us to precisely determine which atoms in the reactants are transformed into specific atoms in the products during a chemical reaction.

**Fig. 1:**
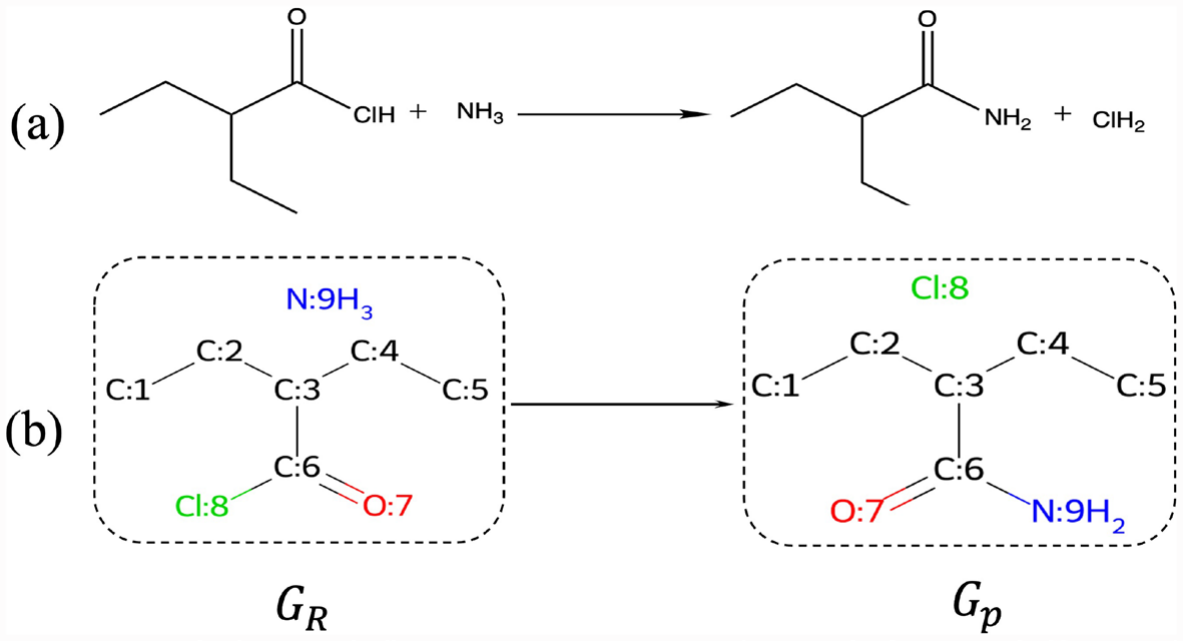
**a** A reaction example; **b** Graphical representation of one possible atom mappings. All hydrogen atoms connected to carbon atoms are omitted to simplify the figure

Graph representation of molecules is a natural way to represent molecules. Figure 1a represents a chemical reaction, and Fig. 1b shows its corresponding graphical representation of the atom mapped reaction.

To construct graphs from molecules, we represent each atom in the molecule as a node, and two nodes are connected if exist chemical bonds between these atoms. Each graph *G*(*V*, *A*, *X*, *E*) is composed of a set of atoms *V*, an adjacency matrix ${A} \in \{0,1\}^{\mid {V}\mid \times \mid {V}\mid }$, an atom feature matrix $X \in {R}^{\mid {V}\mid \times \mid {N_F}\mid }$, and a bond feature matrix $E \in {R}^{\mid {V}\mid ^2 \times \mid {E_F}\mid }$; where the length of the atom feature and bond feature are denoted by $|N_F|$ and $|E_F|$, respectively.

To establish a precise correspondence between atoms in the reactant and product molecules, we define a mapping function *M* that assigns a unique label to each atom in the reactant molecules $G_R \left( {V}_R, {A}_R, X_R, E_R \right)$, such that the corresponding atom in the product molecules ${G}_P\left( {V}_P, {A}_P, X_P, E_P\right)$ receives the same label, $\text {M}: V_R \rightarrow V_P$. This mapping function *M* ensures that each atom in the reactant molecules is uniquely mapped to a corresponding atom in the product molecules, preserving connectivity and atom types. We represent this mapping using a binary correspondence matrix denoted as $M \in \{0,1\}^{\mid V_R \mid \times \mid V_P \mid }$, where $M[i, i^\prime ] = 1$ if node *i* in the reactant graph corresponds to node $i^\prime$ in the product graph and 0 otherwise.

However, many molecules are symmetric, leading to the possibility of multiple valid atom mappings for a single reaction. Identifying atoms with the same chemical environment and identical properties is essential for atom mapping tasks. Essentially, the presence of these atoms, known as topologically equivalent atoms, introduces additional complexity to atom mapping tasks when multiple valid mappings are possible. For example, in Fig. 1, the carbon atoms 1 and 5, as well as 2 and 4 are topologically equivalent. As a result, four distinct possible atom mappings can be derived:

i. $$1 \rightarrow 1, 2 \rightarrow 2, 4 \rightarrow 4, 5 \rightarrow 5$$
ii. $$1 \rightarrow 5, 2 \rightarrow 2, 4 \rightarrow 4, 5 \rightarrow 1$$
iii. $$1 \rightarrow 1, 2 \rightarrow 4, 4 \rightarrow 2, 5 \rightarrow 5$$
iv. $$1 \rightarrow 5, 2 \rightarrow 4, 4 \rightarrow 2, 5 \rightarrow 1$$

In this example, mappings ii and iii are less favorable than mappings i and iv since they introduce additional bond edits. However, the challenge arises from the fact that no atom mapping method can definitively determine whether to map $1 \rightarrow 1$ or $1 \rightarrow 5$ (i and iv), leading to ambiguity in selecting the correct mapping.

### Learning graph matching

Learning graph matching involves the process of developing models that can predict matches between pairs of nodes in two graphs based on data. These models utilize node features to extract relevant information for matching and apply learned knowledge to new graph matching problems.

In the context of deep graph matching methods, the core concept revolves around creating an end-to-end learning model. This model aims to extract meaningful affinities from graphs using differentiable optimization techniques. A key tool in achieving this goal is the utilization of Graph Neural Networks (GNNs), well-suited for handling graph-structured data and capturing intricate relationships between nodes [32]. GNNs empower us to efficiently determine the mapping between reactant and product atoms, thereby facilitating precise atom mapping in chemical reactions.

GNNs are a class of neural networks designed specifically for learning from graph-structured data. Unlike traditional neural networks that operate on fixed-dimensional data such as images and sequences, GNNs can handle data represented in the form of graphs. The power of GNNs lies in their ability to capture complex relationships and dependencies between nodes in a graph.

In GNNs, neighboring nodes interact and exchange information iteratively through message passing. This information typically includes node features, edge features, and the adjacency matrix. Node features are gathered in a matrix containing features representing each node in the graph. In the context of molecular graphs, these features could include information about the atom type and atomic properties. Similarly, the edge features matrix contains features representing the edges in the graph. These features could include information about bond properties such as bond type, bond length, etc. The adjacency matrix, on the other hand, is a binary matrix representing the connections between nodes (atoms) in the graph. The entry (*i*, *j*) in the adjacency matrix is 1 if there is an edge between node *i* and node *j* and 0 otherwise.

The message passing process in GNNs involves updating node features at each step by aggregating information from each node *i* and its neighbors, denoted by *j*, as shown in Eq. 1:

1 $$\begin{aligned} {h}_{i}^{(t)} = {\text {update}}( {h}_{i}^{(t-1)}, {\text {aggregate}}( {h}_{i}^{(t-1)}, {h}_{j}^{(t-1)}, e_{ij}^{(t-1)})), \end{aligned}$$

where ${h}_{i}^{(0)}$ and $e_{ij}^{0}$ are the initial node feature and edge feature, respectively. Index *j* belongs to the set of neighbors of the node *i*. The ${\text {update}}$ is a differentiable function, and ${\text {aggregate}}$ is a permutation invariant operator. Various aggregation and updating functions can be applied, including mean, max, and sum.

By repeatedly applying the message passing process for several steps, GNNs effectively learn to encode both the graph structure and node features into meaningful embeddings. Therefore, these node embeddings encapsulate valuable structural and semantic information, making them highly effective for graph comparison and matching tasks based on their learned representations.

Various neural architectures have been proposed to address the task of graph matching and graph similarity by learning from data. Some methods focus on comparing whole graphs to identify graph similarity such as [28, 33, 34]. On the other hand, some methods are designed to work by matching nodes, mainly for the purpose of graph matching, like what’s discussed in references such as [26, 35, 36].

### Identifying topologically equivalent atoms with Weisfeiler-Lehman test

Topologically equivalent atoms are atoms within a molecule that have the same chemical environment and exhibit identical properties in a given chemical context. In other words, topologically equivalent atoms share the same connectivity and bond arrangement with their neighboring atoms, leading to similar chemical behaviors. By recognizing these topologically equivalent atoms, we can overcome atom mapping ambiguities and ensure accurate correspondence between reactants and products, particularly in complex reactions involving large, symmetric molecules.

In this study, we utilize an adaptation of the Weisfeiler-Lehman (WL) test for identifying topologically equivalent atoms within a molecule. The WL test is an algorithm used for graph isomorphism testing [37]. The WL algorithm works by iteratively refining the labels of the nodes in the graph based on the neighborhoods of each node. During each iteration, the algorithm computes a hash of each node’s neighborhood and assigns the hash as a new label to that node. This process is repeated for a predetermined number of iterations. The final labelings for both graphs are then compared, and if they are identical, it indicates that the graphs are likely isomorphic.

We consider two atoms to be topologically equivalent if they have the same atomic symbol and their three hop neighbors are the same. In contrast to [24], topologically equivalent atoms are defined as those of the same element, connected to the same atom, and not connected to any other atom. Further details of this identification process are available in Appendix A.

**Fig. 2:**
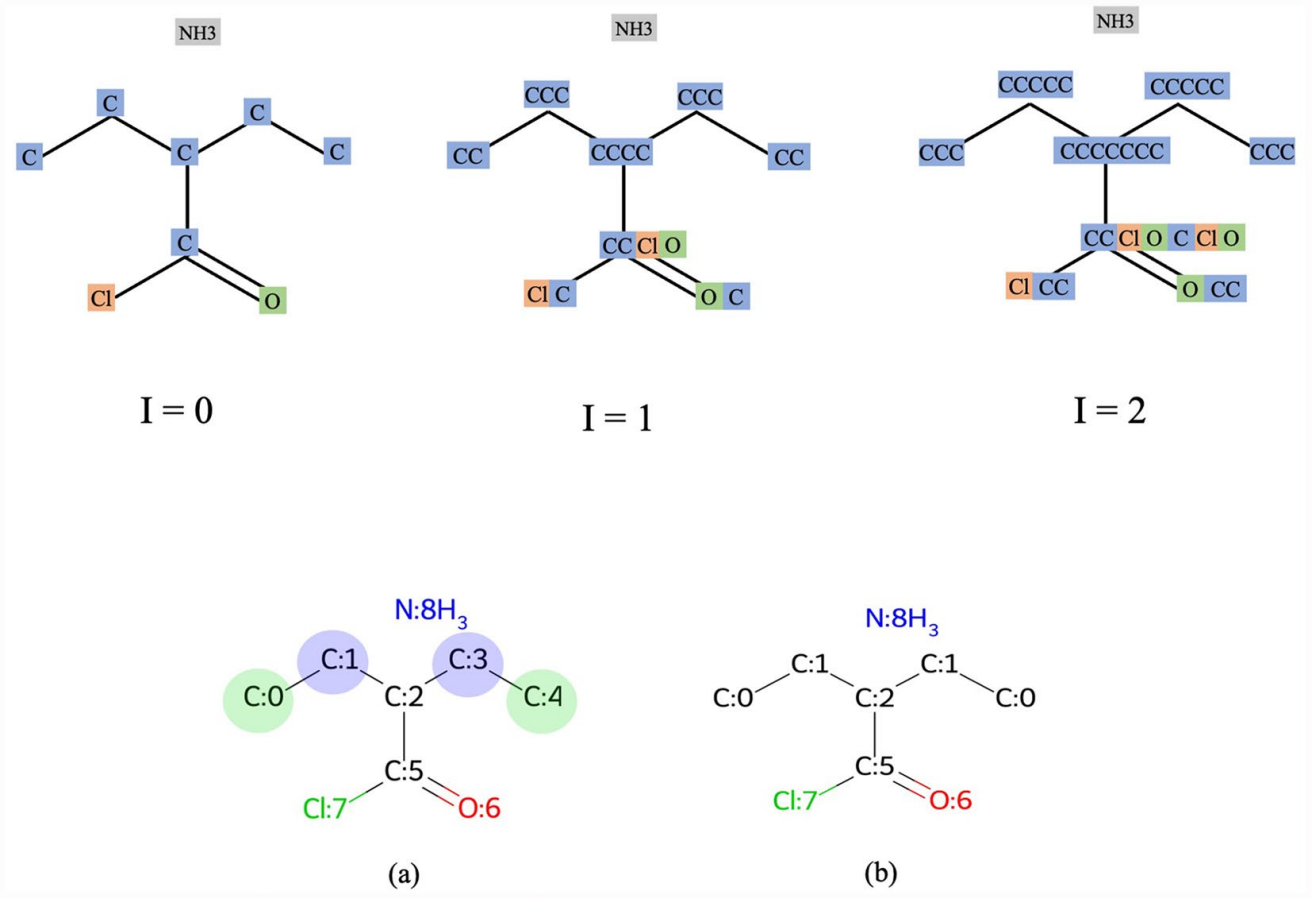
Top: three iterations of the adapted WL test, showcasing the progressive augmentation of node labels. Bottom: **a** An example of a molecule with symmetry in which carbon atoms colored with the same color are topologically equivalent; **b** Detected topologically equivalent atoms by our proposed WL test

**Fig. 3:**
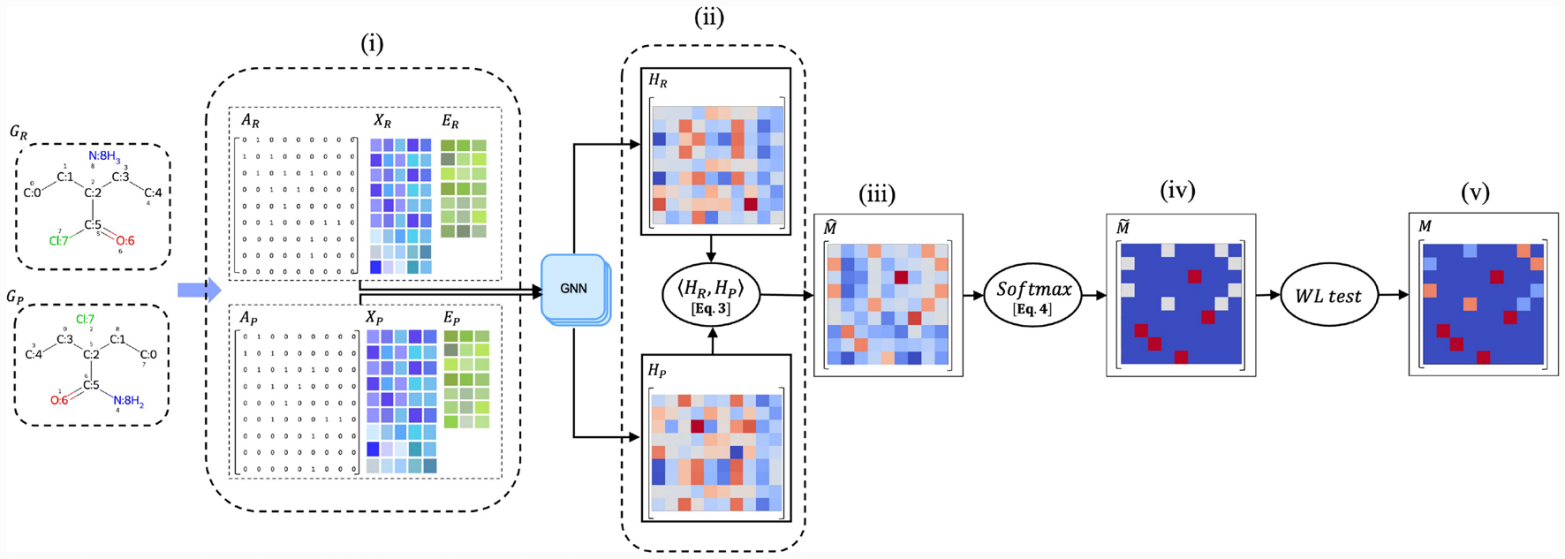
Workflow of AMNet for atom mapping: AMNet utilizes a combination of feature matching, molecular symmetry identification, and correspondence refinement to establish correspondences between atoms in the reactant graph and product graph. The process involves: (i) Transforming molecular structures into graphs and generating node embeddings to capture structure and features. (ii) Pairwise matching scores are computed between reactant and product embedded graphs, (iii) yielding the initial correspondence matrix. (iv) Normalizing this matrix provides matching probabilities. (v) Symmetry identification by the Weisfeiler-Lehman test

Figure 2 illustrates the process of identifying molecular symmetry using the WL test. In the initial step ($I=0$), atoms have their actual atomic symbols. Subsequently, in step $I=1$, neighbor atomic symbols are augmented for each atom. In the subsequent iteration, denoted as $I=2$, the process is further illustrated in the figure. This iteration represents the next step in the WL test, where node labels are refined based on the augmented information from the neighborhoods. In this example, after one iteration, topologically equivalent atoms can be identified. Figure 2 bottom visually represents the successful detection of topologically equivalent atoms by our proposed WL test. In Fig. 2b, carbon atoms sharing the same color are topologically equivalent, and Fig. 3c shows that our adapted WL test provides the same atom mapping number for topologically equivalent atoms.

After applying the Weisfeiler-Lehman test and detecting topologically equivalent atoms within the molecular graph, we organize this information into sets to leverage it during the network training process. Each set represents a group of topologically equivalent atoms within the molecule. Specifically, a set will contain at least one element if there are no other topologically equivalent atoms present in the molecule. On the other hand, if there are multiple topologically equivalent atoms in the molecule, the set will include more than one element.

### Atom matching network

In order to find a correspondence between two molecular graphs, we proposed a graph-based neural network architecture. This model, which we named Atom Matching Network (AMNet), aimed to provide efficient atom mapping solutions. Figure 3 illustrates the workflow of AMNet. The process consists of multiple steps involving graph generation, symmetry identification, and feature matching.

The initial step involves transforming molecular structures into graphs, incorporating atom and bond features that encapsulate their distinctive attributes. The molecular graph is then processed by Graph Isomorphism Networks (GIN) [38]. GINs are a type of graph neural network that is particularly effective in capturing complex relationships between nodes. GIN enables the transformation of each node within the input molecular graph into an embedding space. These node embeddings capture both the topological structure of the nodes and their features.

To achieve this embedding, a shared weight neural network, represented by GNN in Fig. 3, takes as input the adjacency matrices of both molecular graphs ($A_R$ and $A_P$), as well as their node features ($X_R$ and $X_P$) and edge features ($E_R$ and $E_P$). Subsequently, this GNN generates node embedding representations of each graph (${H}_R$ and ${H}_P$ for the reactant molecular graph and the product molecular graph, respectively).

2 $$\begin{aligned} {H}_R&= \text {GNN}( {A}_R, X_R, E_R), \nonumber \\ {H}_P&= \text {GNN}( {A}_P, X_P, E_P). \end{aligned}$$

This process brings both molecular graphs into the same space; therefore, pairwise matching scores can be computed between the nodes of ${G}_R$ and ${G}_P$ using a similarity function (e.g., dot product), which takes as input the features of two vectors, and its output is a scalar similarity score. These pairwise matching scores are stored in the initial correspondence matrix ${\hat{M}}$. Each element ${\hat{M}}_{i,i^{\prime }}$ of the matrix corresponds to the matching score between the $i-th$ node in ${G}_R$ and the $i^{\prime }-th$ node in ${G}_P$.

3 $$\begin{aligned} {\hat{M}} = \langle {H}_R,{H}_P\rangle . \end{aligned}$$

Then, to obtain the pairwise matching probabilities, we normalize the matrix ${\hat{M}}$ row-wise. The normalized matrix ${\tilde{M}}$ has entries given by:

4 $$\begin{aligned} {\tilde{M}}_{i,i^{\prime }} = \frac{exp({\hat{M}}_{i,i^{\prime }})}{\sum _{k=1}^{|v_R|} exp({\hat{M}}_{k,i^{\prime }})} , \end{aligned}$$

where $$i \in V_R$$ and $$i^{\prime } \in V_P$$.

In other words, the matrix ${\tilde{M}}$ can be interpreted as a correspondence matrix that assigns a probability to each pair of nodes in ${G}_R$ and ${G}_P$, indicating the likelihood of each node in ${G}_R$ being matched with each node in ${G}_P$.

Then, to avoid penalizing the model for failing to distinguish between topologically equivalent atoms, we take advantage of molecular symmetry information explained in Sect. 2.3. We apply the WL test to ${\tilde{M}}$ to obtain *M*. This approach recognizes the inherent symmetry and allows the model to focus on distinguishing between non-topologically equivalent atoms, resulting in a more efficient and accurate atom mapping process.

We train the model using ground truth correspondence matrices, which are matrices indicating that atom index *i* in the reactant corresponds to atom index *i* in the product. This ground truth matrix is referred to as $\pi _{\textrm{gt}}(\cdot )$. Throughout the training process, our objective is to minimize the negative log-likelihood of correct correspondence scores, as depicted by Eq. 5.

5 $$\begin{aligned} {\mathcal {L}}=-\sum _{i \in {V}_{R}} \log (M_{i, \pi _{\textrm{gt}}(i)}) \text {.} \end{aligned}$$

## Experiments

### Setup

**Data**: To determine how well our proposed model can identify the atom correspondence between reactants and products, we analyzed 15,000 reaction examples obtained from [1]. This dataset was sourced from the United States Patent and Trademark Office (USPTO) reaction data [39]. Each line in the dataset includes the reaction SMILES string and four types of reaction edits (atoms that lost hydrogen, atoms that obtained hydrogen, deleted bonds, and added bonds). The model was trained, validated, and tested using 70%, 10%, and 20% split of the data, respectively. We aim to compute the atom mappings for all nonhydrogen atoms.

In this dataset, on the product side, reagents and catalyzers are excluded. To balance reactions, meaning that the number and types of atoms on the reactant side are identical to those on the product side, we construct products by applying reaction edits to the reactants. Reaction edits involve modifying the structure of the reactant graphs to create product graphs. After constructing the products, we first validate them by checking for valence constraints and then compare the main components of the generated products with the original products from the dataset. As a result, atom indices within the reactants and products are aligned with their corresponding atom mapping numbers within the dataset. This characteristic potentially leads to predictions being overly optimistic due to their reliance on atom positions. To mitigate this issue, we remapped reactions in the dataset to eliminate atom position dependence. Further details of this process are available in Appendix B.

*Feature extraction* In order to generate graphs from the molecules, a wide range of atom and bond features are used. These features are computed using the RDKit open-source package and are represented as one-hot encodings. These one-hot encoded features are concatenated to create a comprehensive representation of the molecular structure. This concatenated feature vector encapsulates detailed information about the atoms and bonds present in the molecule, allowing the model to capture and analyze the intricate characteristics of the molecular structure effectively. Tables 1 and 2 detail the atom features and bond features, respectively. The “Size” column in Tables 1 and 2 represents the dimensionality of each one-hot encoded feature vector.

**Table 1:** Atom Features

Feature	Description	Size
Atom Type	Atom type	64
# Heavy Neighbors	0, 1, 2, 3, 4, More than four	6
Formal Charge	-3, -2, -1, 0, 1, 2, 3, Extreme	8
Hybridization	s, sp, sp2, sp3, sp3d, sp3d2, Other	7
Explicit Valence	1, 2, 3, 4, 5, 6	6
Is In Ring	Whether atom is part of a ring	1
Aromaticity	Whether atom is part of an aromatic group	1
Atomic Mass Scaled	Normalized atom mass	1
VDW Radius Scaled	Normalized van der waals radius	1
Covalent Radius Scaled	Normalized covalent radius	
Chirality Type	Unspecified, Tetrahedral CW, Tetrahedral CCW, Other	4
# Hydrogen	0, 1, 2, 3, 4, More than four	6

**Table 2:** Bond Features

Feature	Description	Size
Bond Type	Single, double, triple, or aromatic	4
Conjugated	Whether the bond is conjugated	1
In Ring	Whether the bond is part of a ring	1

*Evaluation* To evaluate the performance of the model, we report the percentages of correctly mapped reactions at the top@1, top@3, top@5, and top@10 and the average accuracy of the prediction on the test dataset. Top@k indicates the number of reactions correctly mapped when the mapped atom is correct in the first top k prediction. The average accuracy of atom mapping is calculated by summing up the accuracy of the predicted atom mapping of each reaction and then dividing it by the total number of reactions in the test set. We assess AMNet across various tasks. In our initial task, our primary objective was to evaluate the effect of identifying molecular symmetry on atom mapping predictions. This experiment involves comparing models that incorporate the identification of molecular symmetry with those that do not. Our second task explores understanding the influence of feature selection on the performance of the AMNet. This step is crucial in understanding how the choice of features impacts the accuracy and overall quality of our atom mapping predictions. For our final evaluation, we employ a subset of the Golden dataset [19], which is widely recognized in the assessment of different atom mapping approaches, to ensure a fair comparison with RXNMapper [20]. The decision not to directly compare AMNet and RXNMapper on the USPTO dataset stems from RXNMapper’s training process, which involved training on the USPTO dataset itself. Given that we partitioned the USPTO dataset into distinct training and testing sets for AMNet, there is uncertainty about whether the subset we used for testing overlapped with RXNMapper’s training data.

*Implementation* Our model is implemented in PyTorch, utilizing the PyTorch Geometric [40] libraries. The implementation process is conducted in parallel on GPUs within a high-performance computing environment. To optimize the model’s performance, we examined various hyperparameter settings. The results indicate an embedding dimension of 512, along with a total of 3 message passing layers, yielded the most favorable outcome. Throughout all experiments, to create a standardized benchmark for comparison, we ensured the hyperparameter settings remained consistent. Optimization is achieved using the ADAM optimizer with a fixed learning rate of 0.0001. To prevent overfitting of the model, we applied the early stopping method to our training process. We employ a strategy known as Jumping Knowledge [41], which is the concatenation of node embeddings from each iteration of the message-passing layer.

### Effect of molecule symmetry identification

In this experiment, we investigated how the identification of molecular symmetry affects atom mapping prediction by comparing models with and without the identification of molecular symmetry.

**Table 3:** Performance of the AMNet with and without molecule symmetry identification

Symmetry	Avg. Acc.	%Top@1	%Top@3	%Top@5	%Top@10
	$(\%)\pm \hbox {std}$	$(\%)\pm \hbox {std}$	$(\%)\pm \hbox {std}$	$(\%)\pm \hbox {std}$	$(\%)\pm \hbox {std}$
Yes	${{\textbf {97.3}}}\pm 0.1$	${{\textbf {66.2}}}\pm 0.1$	${{\textbf {96.6}}}\pm 0.0$	${{\textbf {99.3}}}\pm 0.0$	${{\textbf {99.7}}}\pm 0.0$
No	$83.7\pm 0.2$	$43.8\pm 0.2$	$79.9\pm 0.1$	$96.2 \pm 0.0$	$98.7\pm 0.0$

The highest average accuracy and Top@k are highlighted in bold font

Table 3 presents the performance evaluation of two models on the USPTO-15k test dataset. The result highlights that the incorporation of molecule symmetry identification significantly enhances the performance of the AMNet model for atom mapping. When symmetry is considered, the model exhibits an average accuracy of 97.3% and predicts 99.7% of reactions correctly when the correct mapped atom is on top@10 of the predicted atoms.

**Fig. 4:**
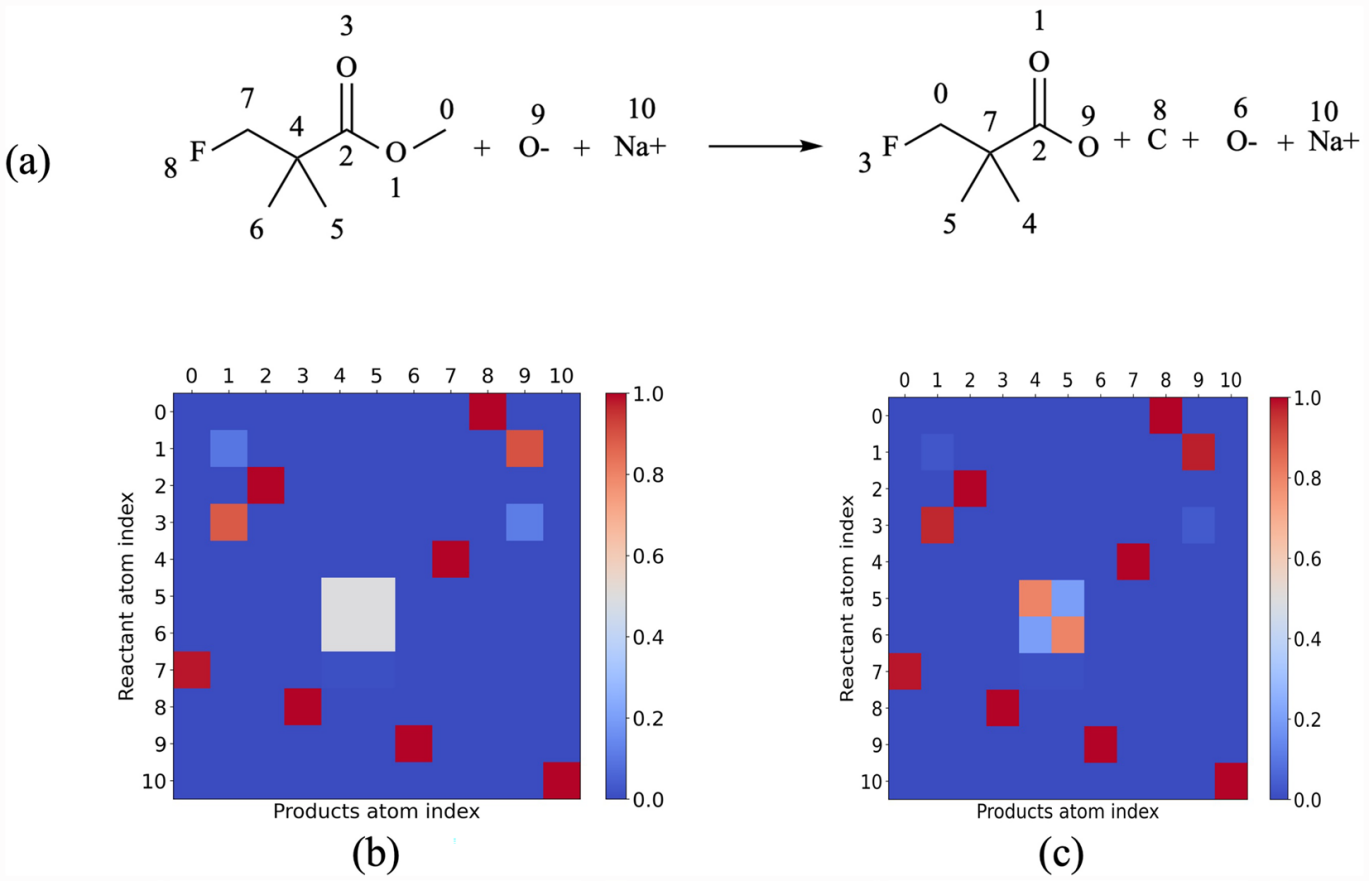
**a** A chemical reaction example, numbers determine atom indices. **b** Predicted correspondence matrix without considering molecular symmetry; **c** Predicted correspondence matrix with considering molecular symmetry. If two atoms are in correspondence, the heat map color is red (the maximum value is one); otherwise, the color is blue (the minimum value is zero). If the model cannot make a decision, it predicts values between 0 and 1. For example, in the case of topologically equivalent atoms, the predictions are orange (atoms 5 and 6 in the reactant)

To enhance our comprehension of how our model predicts atom correspondence, we provide an illustrative example in Fig. 4. This example illustrates a mapped reaction along with the corresponding predicted matrices. Without considering symmetry, the model struggles to distinguish between potential mappings. However, with symmetry identification, the model resolves ambiguity by recognizing equivalent atoms and selecting one correct mapping from two possibilities. As can be seen from this example, it becomes evident that the correspondence matrix predicted without symmetry identification exhibits some degree of uncertainty in its predictions (Carbon 5,6 in reactant and Carbon 4,5 in product).

### Investigation of feature selection impact

In the second experiment, we examined how various atom and bond features affect the performance of the model. Specifically, we aimed to determine how distinct combinations of atom and bond features can impact the atom correspondence prediction. We selected various atom features from Table 1, coupled with the option of including or excluding certain bond features.

**Table 4:** Performance of the AMNet using various choices of features on USPTO-15k test set

Selected	Avg. Acc.	%Top@1	%Top@3	%Top@5	%Top@10
Atom feature	$(\%)\pm \hbox {std}$	$(\%)\pm \hbox {std}$	$(\%)\pm \hbox {std}$	$(\%)\pm \hbox {std}$	$(\%)\pm \hbox {std}$
Whole	${{\textbf {97.3}}}\pm 0.1$	${{\textbf {66.2}}}\pm 0.1$	${{\textbf {96.6}}}\pm 0.0$	${{\textbf {99.3}}}\pm 0.0$	${{\textbf {99.7}}}\pm 0.0$
Whole - Atom type	$47.3\pm 0.5$	$0.6\pm 0.4$	$4.5\pm 0.7$	$12.9\pm 0.7$	$27.4\pm 0.6$
Whole - # heavy neighbors	$97.2\pm 0.1$	$60.4\pm 0.1$	$92.2\pm 0.0$	$98.1\pm 0.0$	$99.6\pm 0.0$
Whole - Formal charge	$97.1\pm 0.1$	$58.1\pm 0.1$	$93.0\pm 0.0$	$98.2\pm 0.0$	$99.5\pm 0.0$
Whole - hybridization	$97.1\pm 0.1$	$59.5\pm 0.1$	$93.1\pm 0.0$	$97.8\pm 0.0$	$99.6\pm 0.0$
Whole - explicit valence	$69.8\pm 0.3$	$1.5\pm 0.3$	$20.8\pm 0.2$	$46.0\pm 0.1$	$77.8\pm 0.0$
Whole - is in ring	$97.2\pm 0.1$	$58.9\pm 0.1$	$93.1\pm 0.0$	$98.2\pm 0.0$	$99.6\pm 0.0$
Whole - aromaticity	$93.0\pm 0.2$	$35.8\pm 0.2$	$72.1\pm 0.1$	$83.2\pm 0.1$	$90.8\pm 0.1$
Whole - atomic mass scaled	$97.2\pm 0.1$	$60.8\pm 0.1$	$93.5\pm 0.0$	$98.1\pm 0.0$	$99.6\pm 0.0$
Whole - VDW radius scaled	$97.2\pm 0.1$	$60.3\pm 0.1$	$93.5\pm 0.0$	$97.8\pm 0.0$	$99.7\pm 0.0$
Whole - covalent radius scaled	$97.1\pm 0.1$	$58.1\pm 0.1$	$93.0\pm 0.0$	$98.2\pm 0.0$	$99.5\pm 0.0$
Whole - chirality type	$40.3\pm 0.4$	$0.4 \pm 0.1$	$2.4\pm 0.4$	$8.1 \pm 0.4$	$25.7\pm 0.3$
Atom type	$95.3 \pm 0.1$	$35.5 \pm 0.1$	$87.7\pm 0.0$	$94.8 \pm 0.0$	$98.9\pm 0.0$
Atom type + aromaticity + explicit valence+ chirality type	$96.4 \pm 0.1$	$61.3 \pm 0.1$	94.1 ± 0.0	98.5 ± 0.0	99.5 ±0.0

The highest average accuracy and Top@k are highlighted in bold font

For each configuration, we trained and assessed the model’s performance using the same set of chosen features. Surprisingly, our findings indicate that the presence or absence of bond features does not have a significant influence on prediction accuracy. One plausible explanation for this observation lies in the architecture of the model itself. Our model utilizes message passing networks, which inherently consider information about neighboring nodes during the prediction process. In doing so, they implicitly incorporate bond information as well. This means that even when bond features are excluded, the model is still capable of capturing some bond-related information through its consideration of neighboring atoms.

The results of experiments on various choices of atom features when excluding bond features are summarized in Table 4. Remarkably, by choosing selected atom features to the “whole” atom features from Table 1, the prediction consistently emerges as the most effective predictor across performance metrics. Notably, excluding essential features, like atom type, severely impacts the model’s performance. The table highlights the significance of specific features. For instance, considering the whole atom features but excluding explicit valence information results in a noticeable drop in accuracy, emphasizing the importance of this feature. Similarly, evaluating atom type along with aromaticity, explicit valence, and chirality type collectively enhances performance.

### Evaluation on the golden dataset subset

To compare the performance of our proposed model with RXNMapper [20], we used the Golden dataset [19], which was originally collected with the aim of benchmarking atom mapping tools. The full dataset consists of 1851 annotated reaction SMILES, for which manually curated atom maps are provided. Our comparison specifically concentrated on a subset of the dataset that contains balanced reactions. Therefore, any conclusions we obtain are specific to this particular atom mapping objective.

RXNMapper initially maps product atoms to reactant atoms, which results in an unwanted permutation of the order of atoms in reactants and products. To compare the predictions by RXNMapper with manually curated data, we standardized the output to remove the effect of this permutation. Further detail of this standardization are available in Appendix C.

**Fig. 5:**
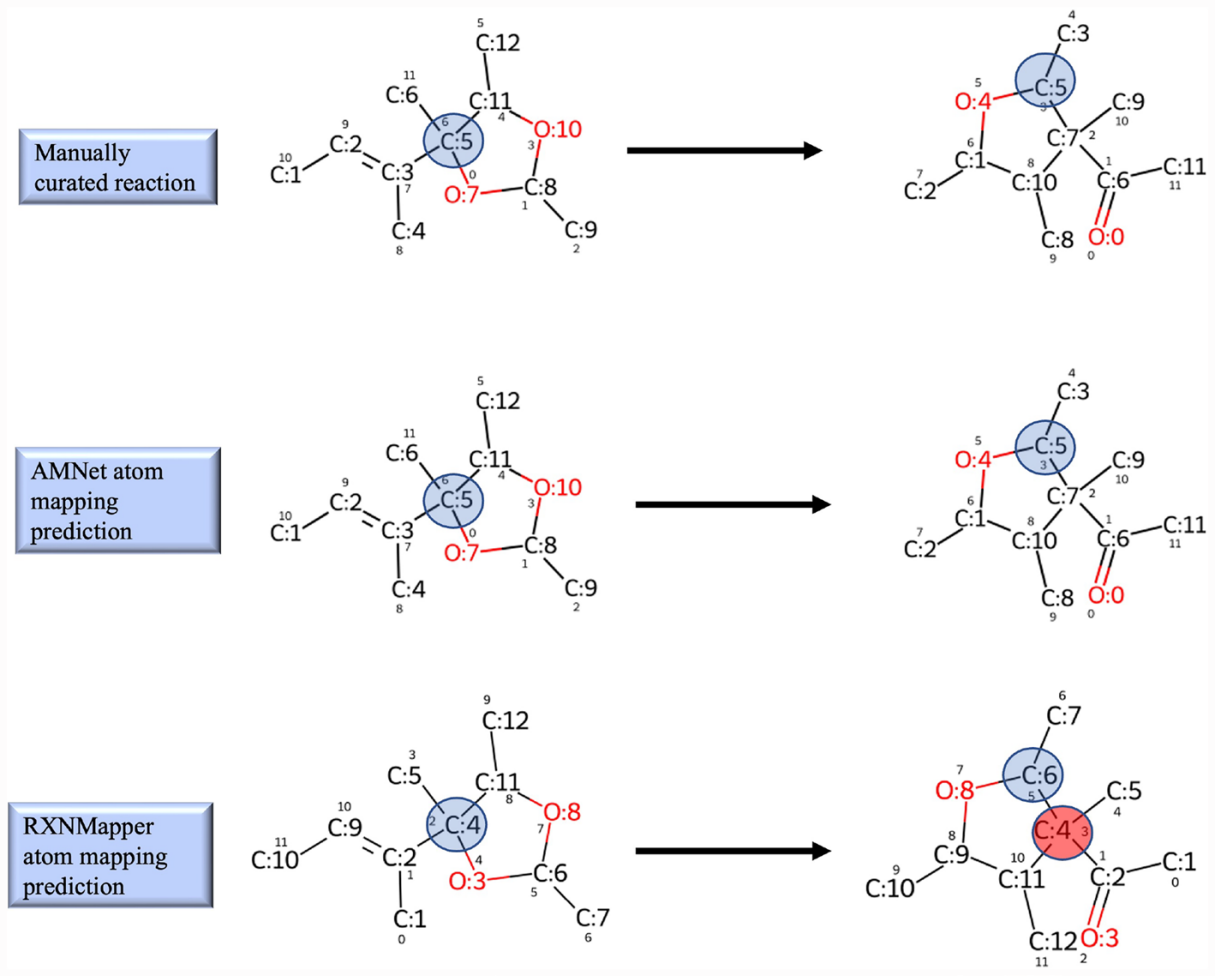
Comparison of a sample reaction where AMNet prediction is matched with ground truth and RXNMapper predicts wrong. Blue circles show the atoms are correctly mapped and the red circle shows the mis-mapping

We assessed the accuracy of a method in predicting atom mappings for a reaction by evaluating the complete alignment of its predicted atom mappings with the ground truth mapped reaction. In other words, a method is considered accurate when the predicted pair atom correspondence can be found in ground truth atom correspondences. Our proposed model achieved an accuracy of 83.3% in atom mapping predictions. The percentage of correctly mapped reactions when the correct atom was mapped by RXNMapper was 79.5%. Figure 5 showcases a scenario where RXNMapper incorrectly predicts atom mapping, while AMNet makes the correct prediction.

### Efficiency assessment and computational complexity

A comparative analysis with existing models highlights notable advantages in terms of training times and hardware requirements. To illustrate, the Graphormermapper, detailed in [23], demanded an extensive 36-hour training period, relying on a sophisticated configuration with 8 NVIDIA A100 GPUs, 40 CPU cores, and 100 GB of RAM. Similarly, Rxnmapper, utilizing the ALBERT model as outlined in [20], required a substantial 48-hour training duration, utilizing a single Nvidia P100 GPU.In contrast, our model demonstrates remarkable efficiency, completing training in just two to three hours using a single GPU and requiring only 20 GB of RAM.

## Conclusion

In this work, we have presented a novel approach to addressing the atom mapping problem in chemical reactions by casting it as a graph matching problem. Our model processes molecular graphs directly, which makes it possible to take advantage of the inherent characteristics of molecules, such as atom and bond properties. The model’s incorporation of symmetry awareness leads to improved accuracy and efficiency in atom mapping. Its end-to-end architecture eliminates the need for prior chemistry expertise, making predictions without any heuristic techniques or post-processing steps. Additionally, the model’s integration of efficient graph matching techniques and deep learning strategies enhances computational efficiency, addressing a common challenge in atom mapping.

In experiments, we systematically explored the effect of molecular symmetry identification and various choices of atom and bond features on model performance. This investigation allowed us to uncover the intricate relationship between feature selection and prediction accuracy. These insights contribute not only to refining our model but also to advancing our comprehension of how specific molecular attributes influence prediction accuracy.

Future work in this research area holds exciting possibilities. Firstly, exploring the application of our model with other datasets beyond the current one will help validate its performance across diverse chemical reactions, potentially uncovering new insights and challenges. Additionally, investigating more complex similarity metrics, such as nonlinear similarity measures, can further refine the model’s ability to identify atom correspondences with higher precision and accuracy.

## Figures and Tables

**Algorithm 1:**
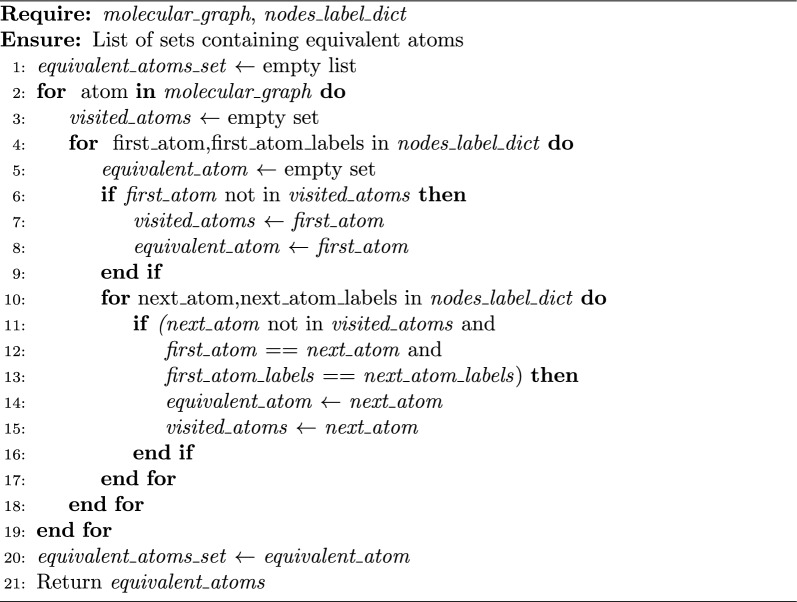
Equivalent atoms identification

**Algorithm 2:**
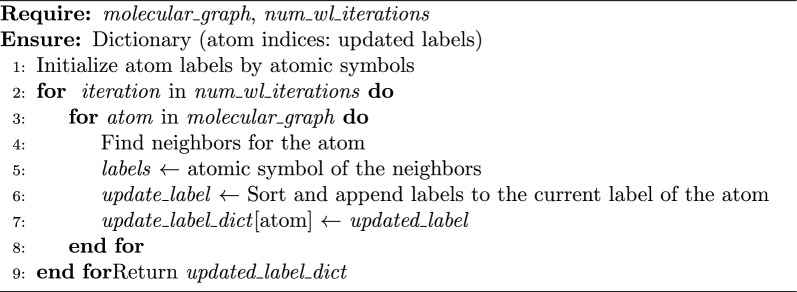
Weisfeiler-Lehman algorithm in one molecule

**Fig. 6:**
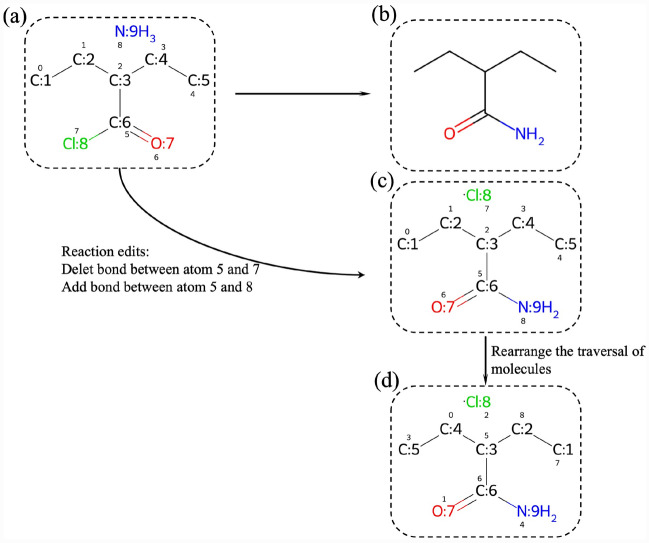
Illustration of an example reaction extracted from the dataset, including the product generation and remapping processes. (a) Original reactant from the dataset; (b) Original product from the dataset, (c) Generated product using reaction edits; (d) Rearranged product. The small numbers in the molecules represent the atom index, while larger numbers show atom mapping index

**Fig. 7:**
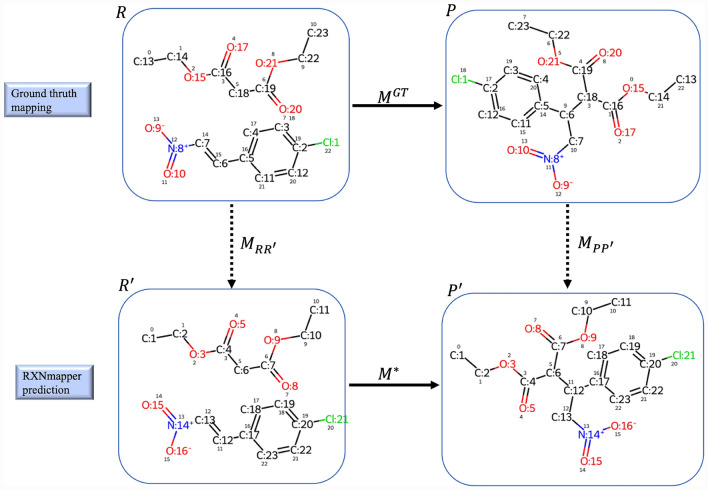
First row: sample reaction of ground truth manually curated reaction, second row: atom mapped reaction by RXNMapper; the small numbers in the molecules represent the atom index, while larger numbers show atom mapping index

## Data Availability

For further reference, the code used in this study is available on GitHub at https://github.com/maryamastero/Atom-matching-network.

## Appendix A

We utilized an adapted version of the Weisfeiler-Lehman test to identify topologically equivalent atoms within a molecule. The criterion for considering two atoms as topologically equivalent is that they have the same atomic symbol and identical three-hop neighbors. Algorithm 1 outlines the process of identifying topologically equivalent atoms.

Algorithm 2 describes the adapted version of the Weisfeiler-Lehman test in one molecular graph. In this algorithm, we initiate the process by initializing atom labels with their corresponding atomic symbols. Subsequently, we iteratively update these labels based on the atomic symbols of their neighbors. This iterative process continues for a predefined number of iterations.

## Appendix B

The dataset is unbalanced as reagents and catalysts are excluded from the product side. Furthermore, atom mapping information is obtained through reaction edits. To guarantee balanced reactions and establish mapping numbers for product atoms, we engaged in the modification of reactants using reaction edits. During this phase, atom indices align with atom mapping numbers. However, this alignment introduces the risk of overly optimistic predictions due to reliance on atom positions, prompting the necessity for a subsequent remapping of reactions to eliminate such dependency. This iterative process ensures a more robust and unbiased representation for predictive modeling. Figure 6 provides a visual representation of an exemplary reaction extracted from the dataset, showcasing the process of product generation through reaction edits and subsequent remapping.

## Appendix C

To compare the prediction by RXNMapper with manually curated data, since RXNMapper permutes the order of atoms in reactants and products, we standardized the output. Figure 7 illustrates an example of a mapped reaction from the Golden dataset and its corresponding atom mapped by RXNMapper. As the reactant and product graphs are isomorphic (depicted as *R* with $R'$ and also *P* with $P'$ in Fig. 7), an exact mapping of atoms in $R \rightarrow R'$ and $P \rightarrow P'$ is achievable. We denote these mappings as $M_{RR'}$ and $M_{PP'}$.

The predicted mappings by RXNMapper and the ground truth mappings are denoted as $M^{*}$ and $M^{GT}$, respectively. For each atom pair *i* in *R* and $i'$ in $R'$, and for each pair of atoms *j* in *P* and $j'$ in $P'$, we establish the relationships: $j \rightarrow M^{GT}[i]$, $j' \rightarrow M^{*}[i']$, $i' \rightarrow M_{RR'}[i]$, and $j' \rightarrow M_{PP'}[j]$. Additionally, we ensure $M_{RR'}[i] \rightarrow M_{PP'}[M^{GT}[i]]$.

It should be noted that, due to molecule symmetry, there can be several matchings from *R* to $R'$ and *P* to $P'$. To consider these possible matches, we define a set of all valid matches in $M_{RR'}$ and $M_{PP'}$.
